# Recombinant 60-kDa heat shock protein from *Paracoccidioides brasiliensis*: is it a good antigen for serological diagnosis of paracoccidioidomycosis?

**DOI:** 10.1590/1414-431X20175928

**Published:** 2017-04-03

**Authors:** G. Peron, F.F. Fernandes, T.N. Landgraf, R. Martinez, A. Panunto-Castelo

**Affiliations:** 1 Departamento de Biologia Estrutural e Funcional, Instituto de Biologia, Universidade Estadual de Campinas, Campinas, SP, Brasil; 2 Departamento de Biologia Celular e Molecular, Faculdade de Medicina de Ribeirão Preto, Universidade de São Paulo, Ribeirão Preto, SP, Brasil; 3 Departamento de Bioquímica e Imunologia, Faculdade de Medicina de Ribeirão Preto, Universidade de São Paulo, Ribeirão Preto, SP, Brasil; 4 Departamento de Clínica Médica, Faculdade de Medicina de Ribeirão Preto, Universidade de São Paulo, Ribeirão Preto, SP, Brasil; 5 Departamento de Biologia, Faculdade de Filosofia Ciências e Letras de Ribeirão Preto, Universidade de São Paulo, Ribeirão Preto, SP, Brasil

**Keywords:** Paracoccidioidomycosis, Heat shock protein, Serodiagnosis, ELISA, Western blotting

## Abstract

*Paracoccidioides brasiliensis* and *P. lutzii* are fungi that cause paracoccidioidomycosis (PCM), the most prevalent systemic mycosis in South America. For serological diagnosis, although 43-kDa glycoprotein (gp43) is regarded as highly specific for PCM, the occurrence of false negative reactions in sera from patients infected with *P. lutzii* suggests that preparation with only one antigen is not recommended. Heat shock proteins are feasible alternatives as a second antigen because they are often highly immunogenic. In this study, we evaluated the usefulness of recombinant 60-kDa heat shock protein from *P. brasiliensis* (rPbHsp60) for the serological diagnosis of PCM. Using western blotting assay, we observed that 77.3% of the sera from PCM patients were positive to rPbHsp60, with 90.9% positivity to recombinant gp43 (rgp43). More importantly, sera from healthy subjects had 27% positivity to rPbHsp60 and none to rgp43. When rPbHsp60 was used in ELISA, we did not observe significant differences between the reactions with sera from PCM patients and healthy subjects, while the difference was clearly evident when the antigen was rgp43. Furthermore, rPbHsp60 was recognized by sera from patients with histoplasmosis, aspergillosis, sporotrichosis or tuberculosis in an ELISA test. These results show that rPbHsp60 is not a good antigen for PCM diagnosis.

## Introduction

The species of dimorphic fungi of the *Paracoccidioides brasiliensis* complex and *P. lutzii* are the etiological agents of paracoccidioidomycosis (PCM) (1), which is a human systemic mycosis highly prevalent in South American countries (2). Brazil has been considered one of the most endemic areas of the world, with about 80% of all active cases of PCM (3).

The polyclonal activation of B cells is a characteristic of PCM that frequently results in hypergammaglobulinemia (4). Although the protective role of antibodies in *P. brasiliensis* infection is controversial, the importance of these molecules in the diagnosis of PCM is undeniable. Moreover, serological assays have proven helpful in clinical monitoring and follow-up of therapy in PCM patients (5).

Many serological techniques have been widely used to evaluate the concentration of antibodies in sera from PCM patients, such as immunodiffusion, counterimmunoelectrophoresis, double immunodiffusion and immunoenzymatic assays (reviewed in Ref. 5). In the last years, assays with single antigens have been more often used than fungal extract comprised of mixtures of antigens. Therefore, many studies have sought to identify and isolate new potential antigens from *P. brasiliensis* for serological tests (6). Hitherto, the 43-kDa glycoprotein (gp43) is unambiguously the most studied *P. brasiliensis* antigen. Besides being one of the most predominant glycoproteins in *P. brasiliensis*, gp43 is recognized by most sera from PCM patients (7).

Because false-negative results have been reported in assays that use gp43 (5), we speculated whether another antigen from *Paracoccidioides* could be used to detect the totality of PCM patients. Among the antigens with potential for use in serodiagnosis of PCM, the heat shock proteins are feasible options, since they are often highly immunogenic and the antibodies against them have been related to the prognosis of many diseases (8).

In 2002, Cunha et al. (9) described a western blot assay using recombinant heat shock protein from *P. brasiliensis* (rPbHsp60) that presented high sensitivity and specificity. They suggested that rPbHsp60 could be used as a single antigen or in association with another one to detect specific antibodies in sera from PCM patients. Thus, in this study, we sought to identify whether rPbHsp60 in association with recombinant gp43 (rgp43) could be useful for PCM diagnosis. We have previously expressed and purified rPbHsp60 and, here, we show the detection of antibodies against rPbHsp60 in the sera from PCM patients. Furthermore, we evaluated the reactivity against rPbHsp60 of the sera from patients with other fungal diseases and tuberculosis.

## Material and Methods

### Serum samples

All patient sera were obtained from the serum bank at the Laboratório de Micologia Médica, Departamento de Clínica Médica, Faculdade de Medicina de Ribeirão Preto, Universidade de São Paulo. In this study, we used 22 serum samples from patients with the chronic form of paracoccidioidomycosis (PCM), 12 with histoplasmosis (H), 12 with aspergillosis (A), 2 with sporotrichosis (S), and 8 with tuberculosis (Tb). Fifteen serum samples from healthy volunteers (without symptoms of infection) were tested as negative control (NC). The use of the sera was approved by the Research Ethics Committee of the Hospital das Clínicas, Faculdade de Medicina de Ribeirão Preto (HCRP), Universidade de São Paulo (protocol HCRP 13982/2005).

### Expression and purification of rPbHsp60 and rgp43

Large-scale expression and purification of rPbHsp60 and rgp43 were prepared as previously described (10).

### Electrophoresis and western blotting

The purified rPbHsp60 and rgp43 were applied to a 12% sodium dodecyl sulfate-polyacrylamide gel electrophoresis (SDS-PAGE) using a Mini Protean Tetra (Bio-Rad, USA). The gel protein bands were stained with Coomassie Brilliant Blue G-250 (Sigma-Aldrich, USA) or transferred to nitrocellulose membranes (Hybond-C Extra, GE Healthcare, USA). Proteins with known molecular weights were used as standards (LMW-SDS Marker Kit; GE Healthcare). Membranes containing 1 µg of rPbHsp60 or 1 µg of rgp43 were blocked with 3% gelatin in Tris-buffered saline (20 mM Tris-HCl, 150 mM NaCl, pH 7.2) containing 0.05% of Tween-20 (TBS-T), overnight at 4°C. Then, these membranes were washed three times with TBS-T and reacted with serum samples from patients with PCM or with serum samples from NC diluted 1:200 in a solution of 1% gelatin in TBS-T, for 2 h at 37°C. After the incubation, the membranes were washed five times and incubated with alkaline phosphatase-conjugated goat anti-human immunoglobulin antibody (isotyped M, G and A – whole molecule; Merck Millipore, USA), diluted 1:10,000 in a solution of 1% gelatin in TBS-T, for 1.5 hours at 37°C. The reaction was developed with BCIP-NBT (5-bromo-4-chloro-3-indolyl phosphate and nitroblue tetrazolium; Sigma-Aldrich), according to the manufacturer's instructions.

### ELISA

The titration of specific antibodies against rPbHsp60 and rgp43 in the sera from patients with PCM was carried out with indirect ELISA. Sera from NC were used for control. Wells of flat-bottomed polystyrene plates (Corning, USA) were coated with 5 µg of rPbHsp60 or rgp43 diluted in 100 µL of 0.2 M carbonate buffer, pH 9.6, and incubated overnight at 4°C. Then, the wells were blocked with 3% gelatin in TBS-T for 1 h and incubated with 100 µL of serum diluted to 1:250 in a solution of 1% of gelatin in TBS-T, at 37°C. After 2 h, the plates were washed five times and the wells were incubated with 100 µL of alkaline phosphatase-conjugated goat anti-human immunoglobulin antibody (Merck Millipore) diluted to 1:2,000, at 37°C. After 1 hour, the wells were washed as described above and the antigen-antibody reaction was detected with a p-nitrophenyl phosphate (pNPP) substrate, according to the manufacturer's instructions (Sigma-Aldrich). The reactions were done in duplicates and the absorbance was determined at a wavelength of 405 nm. The cut-off limit was determined by receiver-operating characteristics (ROC) analysis and was defined as 0.68 absorbance at 405 nm.

### Statistical analysis

Results are reported as the mean values of duplicates. The non-parametric Mann-Whitney test was used to compare two groups, while one-way ANOVA with the nonparametric Kruskal-Wallis test, followed by Dunn's multiple comparison post test, was used to compare the difference among multiple groups. The correlations between the serum reactions with rgp43 and rPbHsp60 were analyzed by means of Spearman's correlation coefficient. Differences were considered to be statistically significant when P<0.05. GraphPad Prism software (USA) was used for statistical analysis. Specificity and sensitivity were analyzed by the ROC curve.

## Results

**Figure 1:**
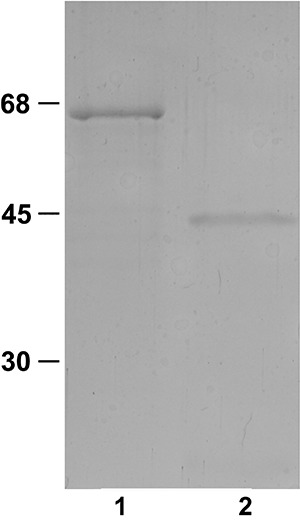
Electrophoresis analysis (SDS-PAGE) of the recombinant proteins gp43 and Hsp60 from *P. brasiliensis*. The electrophoretic migrations of the proteins with known molecular weights are indicated to the left of the figure (shown in kDa). The expected molecular weights for rPbHsp60 (∼60-kDa) and rgp43 (∼43-kDa) bands were observed in the *lanes 1* and *2*, respectively.

To detect anti-Hsp60 and anti-gp43 antibodies in sera from patients with PCM, we produced His-tagged rPbHsp60 and rgp43 in a heterologous bacterial system. Electrophoresis analysis of these recombinant proteins purified in a HisTrap column revealed bands with MW about 60-kDa and 43-kDa, which is consistent with the expected MW for rPbHsp60 and rgp43, respectively (Figure 1).

**Figure 2:**
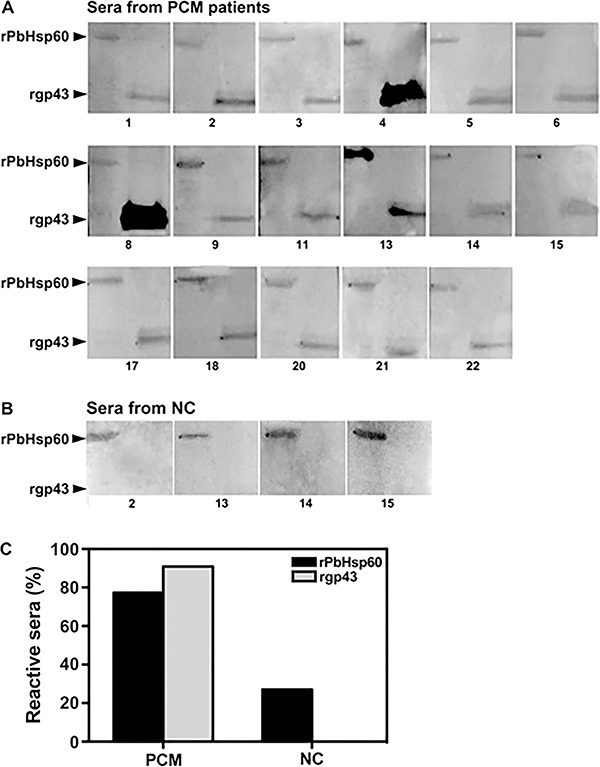
Western blot analysis of the recombinant proteins from *P. brasiliensis* recognized by sera from patients with paracoccidioidomycosis (PCM) or healthy subjects (NC). Recombinant proteins were separated by SDS-PAGE and transferred to nitrocellulose membranes. Membranes containing both proteins were incubated with sera from PCM patients (*A*) or NC subjects (*B*). After incubation with alkaline-phosphatase-conjugated antibody to human immunoglobulin, the reactions were revealed with BCIP-NBT. The arrowheads indicate the migration position of rPbHsp60 and rgp43. The identification of each patient serum is depicted by the numbers below each image. The negative reaction for both proteins is not shown. *C*, Relative number (%) of positive reactions with serum samples from PCM patients and NC group against rPbHsp60 and rgp43. No NC serum was reactive to rgp43.

When these recombinant proteins were used in a western blot assay to detect specific antibodies in the sera from PCM patients, we observed that 17 of 22 sera (77.3%) recognized the rPbHsp60, whereas 20 of 22 (90.9%) reacted with the rgp43 (positive control). To determine if the serological reactions with rPbHsp60 and rgp43 were specific, 15 NC sera were assayed, of which four (27%) reacted with rPbHsp60 (Figure 2B and C), whereas no NC serum was reactive to the rgp43 (Figure 2C). Only the positive reactions were shown in Figure 2A and B.

**Figure 3:**
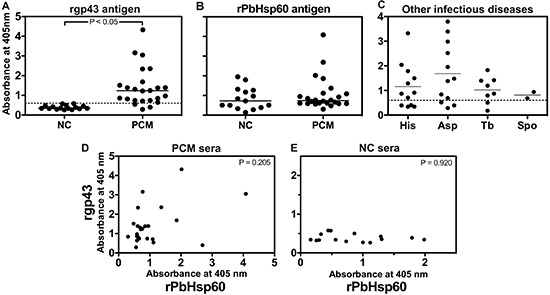
ELISA measurements of anti-gp43 and anti-Hsp60 antibodies from serum of patients with paracoccidioidomycosis (PCM), with other diseases or healthy subjects (NC). Polystyrene plates were coated with (*A*) rgp43 or (*B*) rPbHsp60 and incubated with sera from patients with PCM or NC group. *C*, rPbHsp60 adsorbed on polystyrene plates was also incubated with sera from patients with histoplasmosis (His), aspergillosis (Asp), tuberculosis (Tb), and sporotrichosis (Spo). Results are reported as the mean absorbance value (at 405 nm) of duplicated experiments. The dashed line represents the cut-off point, which was established by the ROC curve, based on the absorbance at 405 nm obtained with sera from PCM patients and NC subjects submitted to reaction with gp43. The full line represents the median absorbance value from each group. Comparison between groups (PCM and NC) was made by non-parametric Mann-Whitney test. Relationship between ELISA measurements of anti-rgp43 and anti-rPbHsp60 antibodies from serum of PCM patients (*D*) and NC subjects (*E*). There was no correlation between the results with rgp43 and rPbHsp60.

Because the western blot tests revealed false positive reactions, we expected and confirmed that those undesirable reactions with sera from NC subjects also occurred when using ELISA, which is used to quantify the antigen-antibody reactions. The cut-off point was established by the ROC curve, based on the absorbance at 405 nm obtained with sera from PCM patients and NC subjects that were submitted to reaction with gp43. A sensitivity of 83.3% and specificity of 100% was determined for a cut-off point of 0.68; absorbance higher than 0.68 was considered to be a positive result. As expected, the titers of the sera from PCM patients with rgp43 were significantly higher than those from NC subjects (Figure 3A) and about 87% of the sera from PCM patients were positive for rgp43. In contrast, many sera from the NC group, such as PCM patient sera, had strong reactivity with rPbHsp60 (Figure 3B). In fact, the immunoassay to detected anti-Hsp60 antibodies from sera revealed a lower specificity (73.33%) and sensibility (77.27%) when compared with anti-rgp43 detection (100 and 90.91%, respectively). Accordingly, the reactions with rPbHsp60 were not significantly different between the sera from NC subjects and PCM patients. There also was no correlation between the reactions obtained with rgp43 and rPbHsp60 to both PCM patients (Figure 3D) and NC (Figure 3E) sera.

When we evaluated the reactivity of the sera from patients with other infectious diseases with rPbHsp60 by ELISA, we observed that most sera were positive to rPbHsp60 (Figure 3C). In contrast, no positive reaction was seen when these sera were assayed with rgp43 (data not shown).

## Discussion

The detection of antibodies against *P. brasiliensis* antigen has been an attractive option for PCM diagnosis and consequently one of the main diagnostic indicators of PCM. As well as permitting rapid inference of the fungal infection, serological diagnosis of PCM has shown to be an important tool for monitoring the treatment of PCM patients (6). In this research, we investigated the reactivity of sera from patients infected with *P. brasiliensis* and other pathogens, as well as healthy subjects, with the antigen rPbHsp60 and showed the presence of a high rate of false positive reactions.

The first aim of this study was to evaluate the usefulness of rPbHsp60 as an antigen for diagnosis of PCM through western blotting assay. Although Cunha et al. (9) have already carried out a similar study with rPbHsp60 for serology of PCM, we found quite different results. By western blotting, they reported that 97.3% of the sera from PCM patients and 9.52% of the normal human sera recognized rPbHsp60, while we found 77.3 and 27% positivity, respectively. Therefore, we obtained a lower reaction positivity rate with sera from PCM patients and a higher rate with serum samples from NC subjects compared with the rates obtained by Cunha et al. (9). The rate of false positivity (27%) suggests that the use of Hsp60 in western blotting for PCM diagnosis is unfeasible.

The second aim of this study was to quantify the antigen-antibody reactions that were initially observed in western blotting, as well as to assess the specificity of rPbHsp60 by ELISA. This latter technique was able to detect high levels of *P. brasiliensis* antibodies in the sera of tested PCM patients. In fact, when we evaluated the reactivity of the sera from PCM patients or NC with rgp43 or rPbHsp60, we found no correlation between the levels of serum antibody and the antigens. Our results also showed that most of the tested sera from patients with other diseases reacted with rPbHsp60. Although these reactions were probably false positive, we do not rule out that they were due to cross-reactivity or a combination of both.

When new antigens are sought for diagnosis, the detection of false positive reactions are cause for concern. Even when the highly specific antigen of *P. brasiliensis*, the gp43, was evaluated for the diagnosis of PCM, the investigators found a percentage of cross-reactivity with sera from patients with other mycosis. Although many factors can contribute to the unspecific reactions, in this case it became clear that the carbohydrate moiety of the gp43 was not a species-specific glycan of *P. brasiliensis* (11–13). The production of recombinant proteins can provide potential solutions for a better diagnosis of PCM. For the gp43, its expression in bacterial systems for heterologous protein, which usually is non-glycosylated, resulted in a molecule that reacted with sera from PCM patients, but not with sera from patients with aspergillosis, candidiasis and histoplasmosis.

In our study, the problem with cross-reactivity is probably linked to homology between the microbial Hsp60, since the analysis of *P. brasiliensis* Hsp60 sequence using Basic Local Alignment Search Tool (BLAST) revealed 93% identity with Hsp60 from *Blastomyces dermatitidis* and *Histoplasma capsulatum*, and 89 and 81% with Hsp60 from *Aspergillus fumigatus* and *Sporothrix schenckii*, respectively (data not shown). We do not consider the possibility that the false positive reactions are related to antibodies directed against the hexa-histidine tag at C-terminus of the rPbHsp60, because no false positivity was seen when we used rgp43, which was produced by using the same expression vector system to produce rPbHsp60, i.e. pET28a. Another problem found was the sensitivity below 100% in the reactions from PCM patient sera with gp43. We cannot exclude that some of our patients were infected with *P. lutzii*, since their sera tend not to react with gp43 (14). In fact, we did not have the identification of the patients’ isolates in species or lineages.

Although Hsp are highly conserved molecules due to their significant role as a chaperone on the folding and unfolding of proteins, the rationale that initially directed our experiments was based on the fact that the Hsp have changed their amino acid sequences or conformational structures during the adaptation to environmental stress in evolving organisms (15). That information can be illustrated by the relative low identity between the amino acid sequences of Hsp60 from *P. brasiliensis* and human, which is about 55% (data not shown). Because Hsp from microbiota can be immunogenic for the host and consequently induce the production of natural or even autoimmune antibodies, anti-Hsp antibodies can be found under normal physiological conditions after exposure to some environmental stress, or may be associated with some autoimmune diseases (8).

Taken together, our results suggest that Hsp60 of *P. brasiliensis* cannot be used to diagnose PCM.
